# Pseudo-méningite inaugurale révélatrice d'une chondrocalcinose articulaire

**DOI:** 10.11604/pamj.2015.20.23.5911

**Published:** 2015-01-08

**Authors:** Mariam Gbané Koné, Hilaire Dossou-Yovo, Mohamed Diomandé, Kouassi Jean-Mermoz Djaha, Baly Ouattara, Boubacar Ouali, Edmond Eti, N'zué Marcel Kouakou

**Affiliations:** 1 Service de Rhumatologie, CHU de Cocody, Abidjan, Côte d'Ivoire

**Keywords:** Syndrome de la dent couronnée, chondrocalcinose articulaire, pseudo-méningite, cracked tooth syndrome-crown, chondrocalcinosis, pseudo-meningitis

## Abstract

Le syndrome de la dent couronnée (SDC) est une étiologie peu connue des cervicalgies aiguës. Il est lié le plus souvent à la chondrocalcinose articulaire (CCA), dont il constitue une des localisations atypiques. L'expression clinique du SDC, à type de cervicalgies fébriles, pose en pratique courante, la problématique d’étiologies diverses dont en particulier les infections susceptibles d’égarer le diagnostic. Nous rapportons un cas de SDC révélé par un tableau de pseudo-méningite.

## Introduction

Le syndrome de la dent couronnée (SDC) se définit par la présence de dépôts de cristaux de calcium (pyrophosphate de calcium ou apatite), dans les structures articulaires de l'odontoïde à savoir la membrane synoviale, capsule articulaire et ligaments [1]. La chondrocalcinose articulaire (CCA) est une arthropathie microcristalline caractérisée par des précipitations de cristaux de pyrophosphate de calcium au sein des cartilages et des fibrocartilages (notamment les genoux, la symphyse pubienne, le poignet) [2], les disques intervertébraux et le ligament transverse de l'atlas peuvent être rarement touchés. Les principales étiologies de ce syndrome sont la CCA (qui survient entre 60 et 70 ans) et le rhumatisme à hydroxy-apatite (40-60 ans) [1, 3] Cette localisation peut revêtir un caractère trompeur et égarer le diagnostic en mimant entre autres, un tableau de cervicalgie fébrile d'origine infectieuse [2]. Nous rapportons le cas d'un patient pris en charge initialement aux urgences médicales pour un syndrome méningé fébrile.

## Patient et observation

Une patiente de 54 ans, sans antécédent particulier, a été transférée des urgences médicales, pour une cervicalgie inflammatoire fébrile avec raideur de la nuque, associée à des céphalées. Aux urgences, le diagnostic de méningite avait été évoqué, une ponction lombaire avait été réalisée, elle avait révélé, une cytologie à 1 élément par mm^3^, une absence de germe bactérien, parasitaire, mycosique et une glycorachie à 0,5 g/L. Malgré ces résultats, la patiente a été mise sous Ceftriaxone 2g par jour en IV pendant deux semaines, et l’évolution n’était pas satisfaisante. Devant la persistance des cervicalgies fébriles, ils évoquent une spondylodiscite et nous l'adressent en rhumatologie. Admise au service de rhumatologie, l'anamnèse révélait en plus de l'atteinte cervicale, une polyarthrite subaigüe, fixe touchant l’épaule droite, les deux genoux, les deux chevilles, les articulations métatarso-phalangiennes et médio-tarsiennes du pied gauche évoluant depuis environ un mois avant l'hospitalisation. Le tout évoluant dans un contexte fébrile avec un amaigrissement de 5 kilogrammes. A l'examen clinique, on notait: Une fièvre à 39°C, une raideur de la nuque accentuée surtout sur les rotations latérales. Le signe de Kernig et le signe de Brudzinski étaient douteux. Il y avait une synovite des deux poignets, du genou gauche et des articulations métatarso-phalangiennes et médio-tarsiennes du pied gauche. L'examen neurologique ne montrait pas de signes déficitaires. La ponction du genou a ramené un liquide inflammatoire trouble.

**Figure 1:**
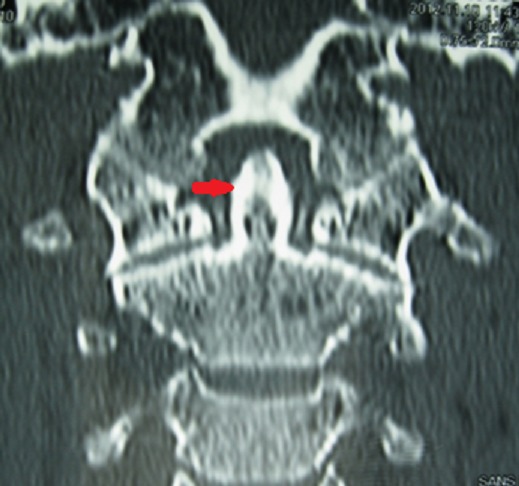
TDM cervicale coronale: calcifications péri-odontoïdiennes (flèche)

**Figure 2:**
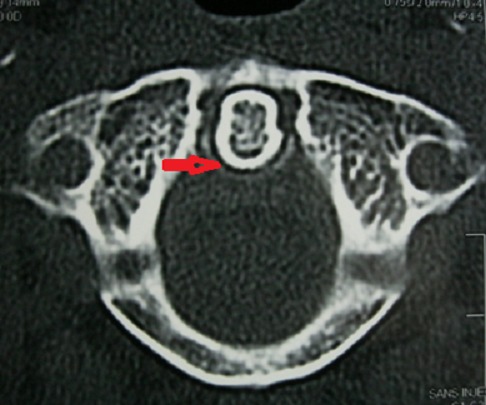
TDM cervicale coupe axiale: calcification du ligament cruciforme en arrière de l'odontoïde, en forme semi circulaire (flèche)

A la biologie, il existait un syndrome inflammatoire (VS à 122 mm à H1, CRP à 110,2 mg/L), il n'y avait pas d'hyperleucocytose à la NFS. Le bilan infectieux (hémocultures, liquide articulaire, ECBU) ne retrouvait pas de germe. Les facteurs rhumatoïdes étaient négatifs et l'uricémie normale. Les radiographies des deux genoux et du bassin révélaient respectivement une méniscocalcinose et un liséré calcique au niveau de la symphyse pubienne. La tomodensitométrie cervicale réalisée en urgence pour éliminer une spondylodiscite, a mis an évidence en C1-C2, une calcification du ligament transverse (en coupes axiales et coronales) (Figure 1, Figure 2), et une calcification du ligament vertébral postérieur (en coupe sagittale), traduisant la dent couronnée. Le diagnostic de SDC en rapport avec une CCA (polyarthrite, liséré calcique aux genoux et au bassin) a été retenu chez cette patiente de 54 ans. Les antibiotiques ont été arrêtés et elle a été mise sous AINS (Diclofénac 150mg/jr per os) et un collier cervical mousse lui a été prescrit. L’évolution a été spectaculaire avec une apyrexie en 48 heures, une régression progressive des cervicalgies et des synovites en quelques jours. La mobilité cervicale était rétablie complètement au bout de 6 mois.

## Discussion

Le syndrome de la dent couronnée a été décrit pour la première fois par Dirheimer et Wackenheim en 1974 [4]. Il se manifeste par un syndrome clinique hétérogène (cervicalgies sensibles aux AINS, raideur cervicale, fièvre) et à la radiographie, on a une calcification du ligament transverse de l'atlas en demi couronne, dense, entourant la partie postérieure de l'odontoïde [2–5]. Il survient le plus souvent chez des femmes [2, 4]. La particularité de notre présentation, c'est son expression clinique trompeuse qui mimait un tableau de méningite infectieuse. En effet devant une cervicalgie fébrile avec une raideur de la nuque et des céphalées, l'urgence diagnostique et thérapeutique reste la méningite infectieuse [2]. Celle-ci va imposer (sauf contre-indication), la pratique d'une ponction lombaire (PL) et la mise en route d'un traitement anti infectieux d'urgence. C'est cette logique qui a certainement amené les médecins urgentistes à mettre un traitement antibiotique quoi que la PL réalisée ne fût pas en faveur de l'infection. Devant la persistance des cervicalgies, ils ont alors reconsidéré leur diagnostic en évoquant cette fois ci une spondylodiscite et nous ont transféré cette patiente. Devant cette pseudo-méningite, l’âge de la patiente, la distribution de l'atteinte articulaire, la découverte de calcifications aux sites préférentiels (genoux, symphyse pubienne et poignet) et grâce surtout à la TDM cervicale nous avons pu «redresser» le diagnostic. La méconnaissance de ce syndrome par le corps médical, pourrait s'expliquer par la rareté de cette affection [1, 2, 4]. C'est à notre connaissance le premier rapporté en Cote d'Ivoire. L’équipement de nos services de soins en techniques d'imagerie sophistiquée a contribué, certainement à l’établissement de ce diagnostic qui est avant tout radiologique. En effet la TDM, constitue l'examen de choix [1, 2, 6, 7]. Elle va mettre en évidence la calcification péri odontoïde semi circulaire qui a donné le nom au syndrome (la dent couronnée). Les coupes axiales et coronales centrées sur C1-C2 sont les plus recommandées [6, 7]. Le diagnostic différentiel se fera, chez un sujet de plus de 50 ans avec une méningite infectieuse, une spondylodiscite cervicale, une polyarthrite rhumatoïde, une tumeur cervicale (métastases de cancers solides ou hémopathies malignes) [2]. Le traitement fait appel aux anti-inflammatoires non stéroïdiens (AINS) et/ou à la colchicine [1, 2, 4, 8], avec une évolution favorable comme constaté chez notre patiente.

## Conclusion

Bien qu’étant rare, le syndrome de la dent couronnée devrait être évoqué devant toute cervicalgie haute, fébrile, pseudo-méningitique chez un sujet âgé. La TDM cervicale en coupe axiale et coronale constitue l'examen de référence pour l’établissement du diagnostic.
